# Nutritional Composition and Health Benefits of Various Botanical Types of Melon (*Cucumis melo* L.)

**DOI:** 10.3390/plants10091755

**Published:** 2021-08-24

**Authors:** Shivapriya Manchali, Kotamballi N. Chidambara Murthy, Vishnuvardana, Bhimanagouda S. Patil

**Affiliations:** 1 College of Horticulture, University of Horticultural Sciences, Gandhi Krishi Vignan Kendra (GKVK), Bengaluru 560065, India; priyachidambara@gmail.com; 2 Central Research Laboratory, Ramaiah Medical College and Hospital, MSRIT Post, MS Ramaiah Nagara, Bengaluru 560054, India; 3 Zonal Horticultural Research and Extension Centre, Sciences Campus, University of Horticultural, GKVK, Bengaluru 560065, India; vishnutiptur@yahoo.com; 4 Vegetable and Fruit Improvement Center, USDA, National Center of Excellence for Melons, Department of Horticultural Sciences, Texas A&M University, College Station, TX 77843, USA

**Keywords:** *Cucumis melo*, ACE inhibition, genotype, landraces, phenotype, phytochemicals, radical scavenging, wild types

## Abstract

Characterizing the diverse melon cultivars for nutrition aids in crop improvement and promoting a healthy diet. Here, we used in vitro assays to characterize the nutritional qualities and health-beneficial effects of 30 melon (*Cucumis melo* L.) genotypes, including 10 improved cultivars, 16 landraces, and 4 wild types collected from different parts of India. Two landraces (Sidoota and Alper Green) had the highest (12.20 and 11.25) total soluble solids (TSS) contents. The Sidoota and Pappusa landraces had high reducing sugar contents (2.84 and 2.81 mg g^−1^ fresh weight [FW]). The highest polyphenols contents (22.0 mg g^−1^ FW) were observed in the landraces Mage Kaayi-2, Budamekaayi, and Small Melon. Reflecting on the primary and secondary metabolite contents, the Mekke Kaayi and Giriyala landraces exhibited high 2,2-diphenyl-1-picrylhydrazyl (DPPH) radical scavenging activity (97.6 and 91% at 100 μg mL^−1^). Additionally, seven of the landraces showed significant nitric oxide (NO) induction activity (>80% inhibition at 200 μg mL^−1^), indicating their potential health benefits, and seven showed considerable angiotensin-converting enzyme (ACE) inhibition activity (highest in Kashi Madhu), indicating their potential usefulness in reducing hypertension. Genotypes with high health beneficial compounds identified in this study can be used for breeding improved melon cultivars to promote these fruits as well as a healthy diet.

## 1. Introduction

Intensive agriculture practices lead to the loss of biodiversity in natural ecosystems and cultivated plants [1]. Maintaining biodiversity is essential for sustainable environments. One of the approaches to maintain biodiversity is identifying crops that are good for the planet and human consumption. Cultivating crops from more natural habitats, such as wild types and landraces, can help to maintain biodiversity [2]. Identifying and characterizing such varieties will help to expand the genetic base for crop breeding and thus play a vital role in modern agriculture. To support this, we characterized a diverse set of melon (*Cucumis melo* L.) cultivars to identify varieties with good nutritional properties. We examined improved cultivars, landraces, and wild types to find genotypes with good quality that may be used for sustainable agriculture by maintaining biodiversity.

Melons are members of the Cucurbitaceae family and include many commercially important crops, encompassing diverse botanical/horticultural types that are widely grown across temperate, subtropical, and tropical regions of the world. A recent report on heterosis nature of melons cultivated in Italy suggested that, among eight genetically distinct breeding lines, correlations of TSS value were significantly different, and it was difficult to predict the outcome based on the genetic background of parents [3]. This work also suggests the significance of breeding line analysis in melons. Melons were domesticated in Asia and Africa for their protein- and lipid-rich seeds [4] and later for their mesocarp or fruit pulp. Human selection developed our current melon varieties from wild melons, which had very small fruit with thin and bitter flesh. The high out-crossing nature of melons has led to different botanical and intermediate forms with different horticultural usages. The recent classification of the species *melo* given by Pitrat (2016) comprises 19 groups and subgroups within the groups [5]. The melons include vegetable types (used for salads, cooking, and pickling), dessert fruit types, and a fragrance type.

Melons of the *agrestis* group are consumed fresh and dried in Africa and Asia [5]. In Africa, oil extracted from the seeds is used for cooking (National Research Council, 2008). In India, in the Rajasthan state, *kachri* melons are cultivated and used as a vegetable [5], but in Southern India (Karnataka and Andra Pradesh), *kachri* melons grow like a weed. These melons are tiny (less than 4 cm long), and the bitter fruits of cultivated *kachri* melons are used in culinary preparations (personal communication). In Sudan, immature fruits of *tibish* melons are used for salad preparation, and some accessions are also cultivated for seeds [5]. Different varieties of *acidulus* melons are cultivated in India and Sri Lanka, and their mature fruits are used for cooking [6]. In India’s southern states, they are widely used as vegetables, and the mature fruits are stored for months by adopting conventional preservation methods. Immature fruits of *conomon*, *flexuosus*, and *chate* melons are used in salads or pickles [5]. The whole fruit is usually consumed in vegetable-type melons. *Momordica* type melons are mainly cultivated in India and Southeast Asia. Ripe fruits are mild in sweetness, and often, the pulp is consumed with sugar or jaggery [7]. Mature fruits of *chandalak* (cultivated from Central Asia to India) and *indicus* (Central and Southern India) are available in sweet [5] and less sweet forms [7]. They are used as a dessert fruit and for juice preparations. Melons of *ameri* (Asia from Turkey to Western China), *casaba* (Western and Central Asia), and *ibericus* (Spain, the Mediterranean area, and in North and South America) varieties are sweet, and the mesocarp is consumed fresh [5]. Honeydew of group *inodorus* and cantaloupe of *cantalopensis* are economically significant because of their high sugar content, strong aroma, and the attractive flesh color (green and orange) and texture of the fruits. External (rind) and internal (pulp) color, shape, and size of the fruit, taste, and aroma have strong effects on consumers’ acceptance of melons. The species *melo* shows extreme genetic variation for fruit traits such as shape, size, presence of netting, sutures and grooves, flesh color, sweetness, consistency, acidity, aroma, sugar composition, etc. [6,8,9,10,11,12,13].

Carotenoids and chlorophylls are the main pigments of melon pulp and rind. Melon fruits also contain protein and sugars; the major antioxidants in melons include phenolic compounds, ascorbic acid, and carotenoids. Seeds contain vitamin E and ω-3 fatty acids [14]. Additionally, melons used as vegetables have flavonoids, alkaloids, and bitter principles, which improve their benefits to human health [15,16]. The method of irrigation and fertilizer supply is known to impact the nutritional quality of melons, as reported by Rolbiecki et al. [17] recently. Therefore, it is important to understand the nutritional variation in cultivated lines. In the present study, the composition of mature fruits representing different botanical groups of melon (cultivated and wild types) was analysed. Further, to understand their potential health benefits, we measured basic health-beneficial components using in vitro assays.

## 2. Results and Discussion

The current study focused on identifying healthy melons for consumption and varieties suitable for use in breeding to obtain more nutritious crops. To this end, we assessed the physical properties (total soluble solids (TSS), color, sugar contents), health-promoting compounds (ascorbic acid, carotenoids, polyphenols), and other health-promoting activities (radical scavenging, NO induction, and angiotensin-converting enzyme (ACE) inhibition) in 30 melon accessions, which included locally available wild types, landraces, and some improved melon varieties. The information generated will help promote the use of locally available melons for health benefits for consumers and help breeders and other researchers use this germplasm for melon improvement.

### 2.1. Selection and Collection of Melon Samples

**Figure 1:**
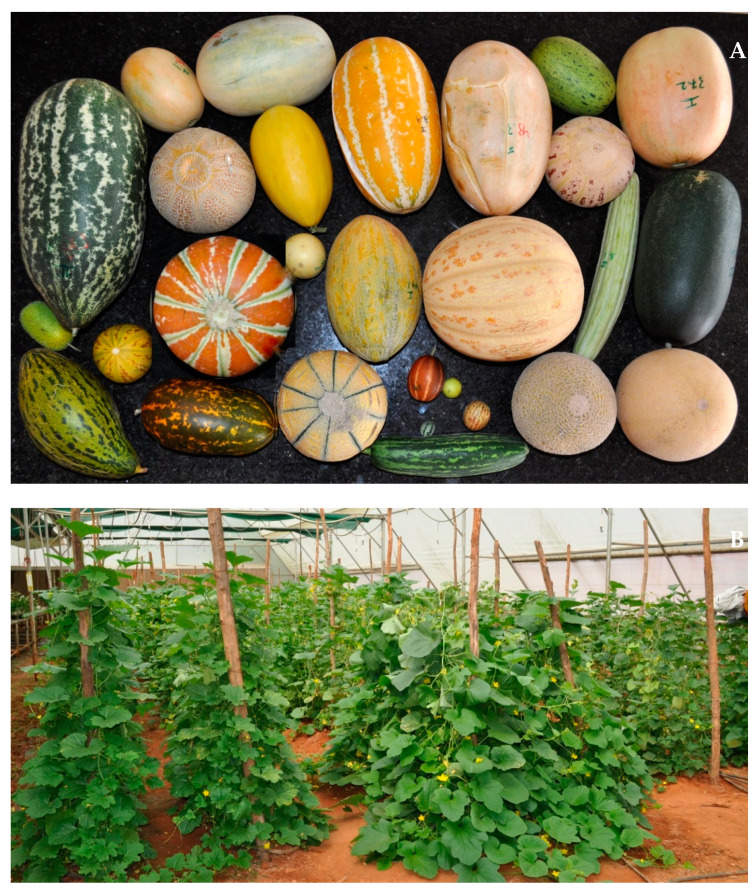
Melons of the various botanical groups used in the study (**A**) and their cultivation under polyhouse conditions (**B**).

Samples of fruits and seeds were collected from different regions of India, and most of the samples from Karnataka (in southern India) were collected personally. Melons from other regions were obtained through national institutes such as The Indian Council of Agricultural Research (ICAR) National Bureau of Plant Genetic Resources, New Delhi; ICAR Indian Institute of Vegetable Research, Varanasi; and ICAR Indian Institute of Horticultural Research, Bengaluru. Details of the botanical group, source, and common use for each sample are given in Table 1. Among the samples, the 10 improved types consisted of 9 dessert fruit types and 1 vegetable type. Among the 16 landraces, 8 are used as fruits/dessert fruit, and 8 are used as vegetables. Among 4 wild types, 1 is used as dessert fruit (Putti Kaayi), and the other 3 are not used. Different samples collected and some of the plants cultivated in our greenhouse used in the current study are as shown in Figure 1.

### 2.2. Measurement of Basic Physical Qualities of Melons

Many parameters can be measured to assess the quality and consumer acceptance of melons. In the current study, we selected total soluble solids (TSS, measured as °Brix (°B)), pH, and titratable acidity as representative of the sweetness, tartness, and mouthfeel of fruit.

Out of 30 samples analyzed, 7 improved varieties and 2 landraces had TSS values of more than 10 °B, with the highest TSS of 12.20 °B found in the landrace Sidoota, followed by the improved melon varieties IC3211356, IC321367, and Kashi Madhu (12.05, 12.0, and 11.25 °B, respectively). The values for improved melons were in the range of 8.15–11.25 °B, except Arka Sheetal, a vegetable-type melon, which had a value of 3.65 °B. In the case of four wild varieties, values were in the range of 3.4–4.20 °B, suggesting most of them were non-sweet melons (Table 2).

Titratable acidity and pH are indicators of the acid nature of fruit pulp, which is known to influence the fruit’s taste. The pH values for melon genotypes ranged from 4.11 (Giriyaala and wild melon) to 7.04 (Cantaloupe 1) with a mean pH of 5.54 (data not shown). Titratable acidity, which is also associated with pH, of fruit pulps was in the range of 0.52% (wild melon) to 0.08% (Cantaloupe 1). Titratable acidity was relatively low in the improved varieties (average of 0.15%), compared with both landraces (average of 0.25%) and wild types (average of 0.24%) (Table 2). The results indicate that lower titratable acidity is more predominant in improved varieties.

To assess sweetness and total carbohydrates in melon, we measured total sugar and reducing sugars using a spectroscopic method (Table 3). The total average sugar content was 38.8 mg g^−1^ fresh weight (FW) with a variation of 11.0 mg. The highest total sugar (61.4 mg g^−1^ FW) was found in the improved variety Cantaloupe 2, followed by the landrace Kekkarike (58.71 mg g^−1^ FW), and the wild type *Agrestis* (54.2 mg g^−1^ FW). The average total sugar content of the wild varieties was 38.7 mg g^−1^ FW with a variation of 12.8 mg g^−1^ FW. The averages of both landrace and improved variety samples were similar with 39.0 mg g^−1^ FW. Reducing sugar, which is directly associated with the sweet taste of the pulp, was in the range of 0.68–2.84 mg g^−1^ FW with a high value of 2.84 mg g^−1^ FW in the Kashi Madhu (improved) and Sidoota (landrace) genotypes. Further, the average reducing sugar content in improved varieties was 2.01 mg g^−1^ FW. Average content of 1.48 mg g^−1^ FW of reducing sugar was observed in the landraces, and 1.08 mg g^−1^ FW in the wild types, indicating most wild types are bitter or non-sweet (Table 3). These physical qualities (TSS, acidity, and sugar content) of fruit indicate the sweetness, taste, and juice content of the fruit, which are critical parameters for consumer acceptance and indicate the shelf life of fruits.

### 2.3. Measurement of Nutritional Components of Melons

**Figure 2:**
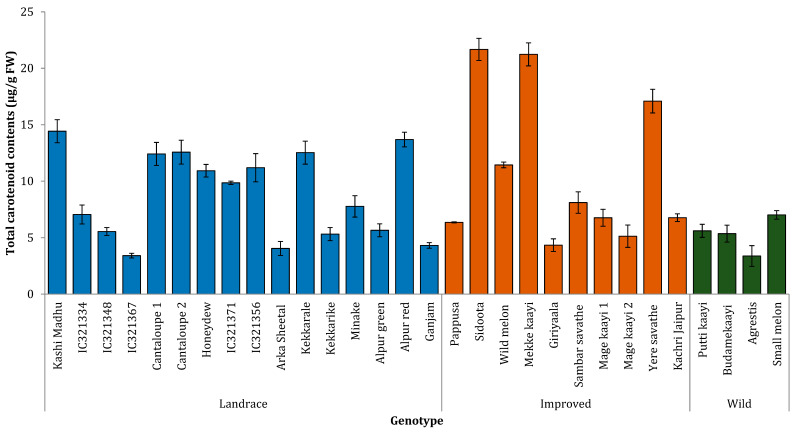
Total carotenoid contents of the melon cultivars as µg per gram of fresh weight (FW). Results are means of three independent experiments and expressed as mean ± SD.

We analyzed the contents of carotenoids, ascorbic acid, and polyphenols to represent the nutritional benefits of melon samples. The average carotenoid content was 9.03 μg^−1^ g FW in all the samples analyzed. Two of the landraces, namely Sidoota (fruit type) and Mekke kaayi (vegetable type), had the highest carotenoid contents with 21.67 and 21.23 μg g^−1^ FW, respectively. Another landrace and locally grown vegetable (Yere savathe) had 17.1 μg g^−1^ FW carotenoids. One of the improved varieties, Kashi Madhu (fruit type), had 14.42 μg g^−1^ FW total carotenoid content. The average carotenoid contents of landraces, improved varieties, and wild type were 9.9, 9.1, and 5.3 μg g^−1^ FW, respectively (Figure 2).

The average ascorbic acid content of thirty samples was 34.5 mg g^−1^ FW, and the highest content was observed in two of the wild varieties, Small melon and Budamekaayi, with 38.2 and 38.6 mg^−1^ g FW, respectively. The ascorbic acid contents did not show significant variation between samples, and all the samples analyzed were good sources of vitamin C (Table 4).

The average for the 30 melon samples analyzed was 12.0 mg g^−1^ FW with variation of 4.13 mg. The highest value was observed in the landrace Mage Kaayi-2 (22.03 mg g^−1^ FW), followed by two wild types, *kachri* and Small melon, which had 18.70 mg g^−1^ FW polyphenols. The average polyphenol content of four wild varieties was 14.35 mg g^−1^ FW, and the landraces and improved varieties had average values of 11.8 and 11.3 mg g^−1^ FW, respectively (Table 4).

To further find the best sources of basic nutrition in the vegetable, fruit, and wild-type melons, and select potential candidates for future cross-breeding and genetic modification, we performed the analysis of variation (ANOVA) of the constituents in these three types and compared the results using the Kruskal Wallis H test. This revealed significant variation between wild and fruit types in the ascorbic acid, polyphenol, and reducing sugar contents (*p* < 0.05) but little difference between the other types (Tables S1 and S2 in Supplementary data).

### 2.4. Measurement of In Vitro Biological Activities of Melons

In the current study, we measured 2,2-diphenyl-1-picrylhydrazyl (DPPH) radical scavenging activity (reflecting antioxidant activity), NO induction, and ACE inhibition to screen melon fruit samples. These assays were performed on whole fruit or pulp juice samples without the addition of water or other ingredients. The DPPH assay was performed at 25, 50, and 100 μg mL^−1^, with ascorbic acid used as the standard, and the results were expressed as % inhibition. All the samples showed dose-dependent increases in theactivity; at 25 μg mL^−1^ DPPH, the average activity was 26.4%, and values ranged from 0.15 to 53.06%. Among improved varieties, Kashi Madhu and IC321334 exhibited the highest activity (89% at 100 μg mL^−1^), and this was significant (*p* < 0.05) compared with ascorbic acid at 25 μg^−1^ mL, which was used as the standard. Among landraces, Mekke kaayi had the highest activity (44.5, 70.8, and 97.6% at 25, 50, and 100 μg mL^−1^, respectively; Table 5). The activity at 100 μg/mL was significant (*p* < 0.05) compared with ascorbic acid at 25 μg mL^−1^. The landrace Giriyaala showed 91% inhibition at 100 μg mL^−1^ and Mage kaayi 2 showed 88.2% activity at 100 μg mL^−1^. Activities of other landraces were moderate. Among wild varieties, Budamekaayi exhibited the highest activity (53, 69, and 80% at 25, 50, and 100 μg mL^−1^, respectively). The small melon activity was similar to Kachri, two Agrestis, and Putti kaayi, which exhibited moderate activity (Table 5).

The trend for NO induction activity was slightly different with the wild variety Agrestis showing the highest activity in comparison with blank (87.2% at 200 μg^−1^ mL and 82.8% at 100 μg^−1^ mL). Following this, Honeydew melon showed 79 and 86% activity at 100 and 200 μg^−1^ mL, respectively. Kashi Madhu and IC321334 showed the best activity among improved varieties along with honeydew. Among landraces, Kekkarale, Alpur red, Ganjam, Pappusa, and Giryala and Yere savathe exhibited more than 80% activity at 200 μg mL^−1^ compared to blank. Among the wild types, the Budame kaayi and Putti Kaayi genotypes had 71.7 and 63.6% activity when compared to blank (Table 6).

**Figure 3:**
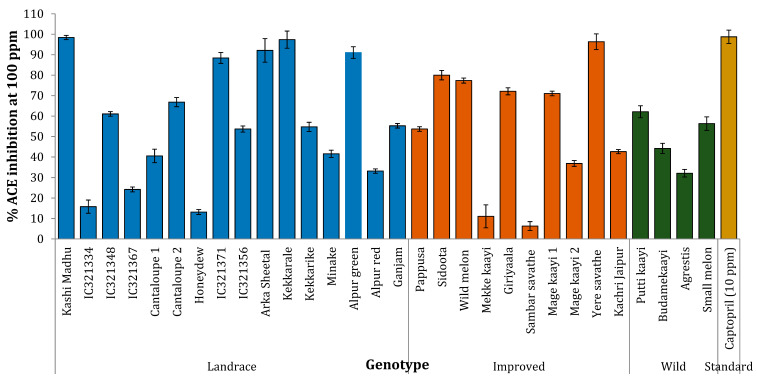
ACE inhibition activity of the melon cultivar flesh samples. Values are calculated from three independent experiments (*n* = 8–9) and are expressed as mean ± SD. Captopril, a known inhibitor of ACE, was used as the standard for comparison, activity was very high at 25 μg mL^−1^ ACE and higher concentrations, and therefore 10 μg mL^−1^ ACE was used for the assay.

ACE inhibition activity (defined as percent inhibition of ACE activity) was significant in many samples in the study. The activity was measured at 100 μg mL^−1^, and the average activity was 55.65% among all the samples. The improved variety Kashi Madhu exhibited the highest activity (98.42%), followed by the landraces Kekkarale (97.4% and 96.6%) and Yeresavathes (96.4%). The landrace Alpur green and the improved variety Arka Sheetal exhibited more than 90% activity. Four wild types were in the range of 32–62%, in this order: Putti kaayi > Small melon > Budamekaayi > Agrestis. Sambar savathe and Honeydew exhibited activity of less than 15% (Figure 3).

## 3. Discussion

Fruits and vegetables provide substantial health benefits, along with basic nutrition. Several clinical, preclinical, and in vitro studies have shown that the consumption of vegetables and fruits protects against chronic diseases [18,19,20,21,22]. This has led researchers and scientists in the agriculture and food sectors to look beyond yields such as bioactive secondary metabolites and nutritional quality when selecting cultivars. It is now mandated to include health benefits as part of quality for all food commodities, including fruits and vegetables. In the current study, we screened 30 melon varieties (Table 1) for their physical properties, primary nutritional parameters, and key secondary metabolites. Further, to explore their potential health benefits, we conducted three in vitro assays on edible portions of these samples.

Melon crops include both fruits and vegetables consumed by humans and consist of a vast range of plants, some bearing sweet and delicious fruits, and some bearing bitter and medicinally important fruits. This variation in morphology and nutritional properties makes them suitable for cultivating customized fruit/vegetables for human consumption. Melons as fruit are sweet and crunchy with an average TSS value of 8–10 °B for the majority of improved varieties [23,24,25]. Our results indicate that among 30 samples, 9 samples had TSS values of more than 10 °B, of which 2 of the improved varieties had TSS of more than 12 °B, and a landrace with 12.20 °B can be considered for sweetness along with high mineral content (Table 2). This landrace is specific to the South Indian region of Karnataka state. TSS is also an indicator of the approximate content of carbohydrates, organic acids, proteins, fats, and minerals.

Another characteristic that determines the sour taste and fruit maturity is titratable acidity. The titratable acidity indicates the contents of all organic acids, including citric and ascorbic acid. The titratable acidity value increases during ripening and with the storage of fruit samples. Lime and lemon have titratable acidity of more than 9%, and pomegranate has a titratable acidity of more than 2% due to its high levels of organic acids [26]. Although citric acid and other organic acid contents are directly proportional to titratable acidity in citrus juice, the content of ascorbic acid is also known to influence the acidity. However, in the melons we tested, there were no correlations between ascorbic acid content and titrable acidity value (correlation value was −0.023) [27]. This could be due to the relatively low content of ascorbic acid and the presence of other organic acids and minerals in melons.

Due to sugar and carbohydrate contents, melons are categorized as low-sugar fruit and release sugar slowly. We measured an average total sugar content of 5.0 g 100^−1^ g of sample in improved cantaloupe varieties (Table 3). Several stress-inducing chemicals enhance the sugar content of melons and other fruits by interfering with carbohydrate synthesis and accelerating ripening. However, getting naturally sweet cultivars will make these fruits more acceptable to consumers, especially when the sweetness is complemented with nutrition and other organoleptic qualities. One of the recent reports also mentioned that among cultivated verities, irrigation and fertilizer influence both yield and the nutritional quality in terms of total sugar, polyphenols, carotenoids, and ascorbic acid content [17]. In addition, there was a difference in the quality of fruits and yield between grafted and non-grafted verities of muskmelon, as observed in one of the recent studies from the United States [28]. Therefore, among the cultivated varieties, use of different agriculture practices is known to influence the quality of fruits and its nutritional quality. Some of the landraces showing more than 50 mg g^−1^ fresh weight of total sugar (accounts for 5 g 100^−1^ g samples) can be considered for breeding to develop sweeter varieties. With a correlation coefficient of 0.62, the majority of samples with high total sugar had significantly higher contents of reducing sugars, which is directly responsible for sweetness. A couple of landraces had more than 2.5 mg g^−1^ of reducing sugar, indicating that they could be good source for developing sweeter melons.

Ascorbic acid, a water-soluble vitamin, can also impart a sour taste to fruits. The content of ascorbic acid in four wild varieties was high, indicating that these can serve as good sources of antioxidants. Melons are also known for good pro-vitamin-A content, mainly in the form of carotenoids. As consumers prefer deep orange and yellow melons, samples of *chandalak*, *indicus*, and intermediate forms of *agrestis* and *kachri*, can be considered for future improvement in carotenoid content (Figure 2). The total polyphenol content is a key indicator of healthful properties in fruits and vegetables [29]. These phenolic compounds include flavonoids, tannins, phenolic acids, and complex phenols. In general, more phenolics accumulate in plants exposed to environmental stress. Our results also indicated that the polyphenol content was highest in wild varieties, at 2.5- to 3-fold more than in improved or landrace cultivars (Table 4). The same could be due to ability of these to fight stress compared to landraces and cultivars.

In order to have a complete picture of the potential health benefits of these fruits, we measured radical scavenging, NO induction, and ACE inhibition ability of melon samples. These activities limit the initiation of many chronic diseases including cardiovascular disease and cancer [30]. Free radicals initiate the breakdown of cellular components, leading to several chronic diseases; therefore, fruits with high radical-scavenging activity hold the potential to decrease the risk of these diseases. Hydrophilic and hydrophobic chemical-based assay scans are used to measure the radical-scavenging ability of samples. DPPH is a rapid, reliable, and commonly used assay to measure hydrophilic radical-scavenging components [31]. Natural compounds with higher DPPH scavenging activity include ascorbic acid, flavanoids, anthocyanin, coumarins, tannins, and other polyphenols [32,33]. Indeed, in our results, DPPH activity was higher in samples with high polyphenols, carotenoids, and ascorbic acid content, indicating that these are major antioxidants in melons (Table 5). Furthermore, our correlation analysis indicated a positive relationship between polyphenols and DPPH activity, along with carotenoid and ascorbic acid content (data not shown). These results were in agreement with the recent report on DPPH activity of differed solvent extracts of *Cucumim melo* L., which reported the highest activity in 100% methanol extract [34].

Nitric oxide induction activity is an indicator of NO donor activity, which is critical in preventing hypertension and cardiac complications [35]. NO donation induces PDE5, which is responsible for the dilation of smooth muscle tissues, including blood vessels, resulting in a reduction in elevated blood pressure [36]. The current study results indicate that some of the melon varieties are sources of NO donation, which may be attributed to carotenoids and polyphenols (Figure 2 and Table 6). Furthermore, some of the amino acid precursors such as L-arginine and L-citrulline are responsible for NO induction activity [36]. In addition to NO induction activity, high water and mineral contents in melon fruits may also help reduce blood pressure.

Finally, we were interested in understanding the effect of melon varieties on inhibition of ACE, which is linked to hypertension and cardio-protection. Polyphenols such as flavonoids, phenolic acids, sterols, and glycosides have significant ACE inhibition activity [37]. Our study results indicated that most melon samples had moderate to high ACE inhibition activity due to polyphenols, carotenoids, and other phytochemicals present in these samples. High activity in landraces and wild varieties (Figure 3) indicate that they can be used to develop healthier melon cultivars.

## 4. Materials and Methods

### 4.1. Chemical and Reagents

All chemicals used in the current study were of analytical grade and procured from SRL Chemical Pvt. Ltd., Bangalore, India. Sodium nitrite, standard ascorbic acid, and carotenoids were purchased from Sigma Chemicals, St. Louis, MO, USA. 2,2-Diphenyl-1-picrylhydrazyl (DPPH), hippuryl-histidyl-leucine (HHL), captopril, and angiotensin-converting enzyme (ACE) were also were purchased from Sigma Chemical Co. All the organic and inorganic solvents used in the study were of analytical grade and were procured from SRL Chemical Pvt. Ltd., Bangalore.

### 4.2. Details of Melon Cultivation and Collection of Samples

The study was carried out from September to December 2018 at the Regional Horticultural Research and Extension Centre, University of Horticultural Sciences Campus, Bengaluru (located at 12°58′ latitude North, 77°11′ longitude East with an altitude of about 930 m above mean sea level), Karnataka State, India. Drip irrigation was used for these plants, and the soil in which they were cultivated was red loam.

The 30 accessions collected for this study included two snapmelon landraces from the *Momordica*, three landraces, and one wild-type accession belonging to the acidulous group. Four accessions (landraces and released varieties) from chandalak, which included both sweet and mildly sweet types from various locations, two accessions from *kachri*, four *indicus* melons and one accession from snake melon (*flexuosus* type), two sweet dessert purpose cultivars of *cantalopensis*, one *inodorous* type (honeydew melon), three *reticulatus*, one each from *agrestis*, *callosus*, and an intermediate form of *agrestis* and *kachri*, and three melons of unknown botanical group (Table 1). The majority of the landraces and weeds used in the study were sampled from different parts of Karnataka state in southern India.

Standard agronomic practices were followed to raise the crop under polyhouse conditions. The average temperature during the crop growth period ranged from 20.3 °C minimum and 31.8 °C maximum to 23.2 °C minimum and 33.8 °C maximum, and the average relative humidity was 91%. Fruits were harvested at appropriate maturation times for each botanical type. Immature fruits of salad-type vegetables, *flexuosus*, and an unknown botanical group were harvested ten days after pollination. The vegetable-type acidulous melon, *agrestis*, and *kachri* were harvested based on a change in rind color from green to yellow. The *momordica* melons were harvested based on the rind color change and fruit splitting. The *inodorus* types were harvested based on a color change in the rind. The *cantalupensis* types were harvested based on the development of netting and color change. The *indicus* and *chandalak* types were harvested based on color change, netting, and aroma. The color of the skin was observed visually, and genotypes were categorized as white, yellow, green, orange, and other. Similarly, the colors of the fruit flesh were recorded as cream, orange, light orange, white, light green, and green.

### 4.3. Fruit Tissue Sampling

The whole immature fruits of vegetable type (salad and whole fruit cooking) melon were cut into small pieces and homogenized in a mixer at low speed to avoid heat generation. In the case of dessert-type melons, the rind was removed, and the flesh was separated and homogenized. These samples were used immediately for analysis of ascorbic acid and carotenoid contents. The remaining samples were stored at −80 °C for further investigation and activity study.

### 4.4. Measurement of Total Soluble Solids

The melon juice was extracted from fruits by pulping. The juice was used to determine total soluble solids by using a handheld refractometer (Atago digital pocket refractometer, 0–53 °Brix). TSS was measured using a standard protocol, and the results were expressed as °Brix [38].

### 4.5. Measurement of Titratable Acidity

The titratable acidity of melon genotypes was determined as previously described [39]. Two grams of pulp was taken in a 50 mL test tube and mixed with distilled water. The pulp was homogenized, volume was made up to 25 mL with distilled water, and a 10 mL aliquot was used for the analysis. This filtered aliquot was taken in a conical flask and titrated against 0.1 N NaOH using 1 or 2 drops of phenolphthalein indicator. The formation of pink color was recorded as the endpoint of the titration. Then, the acidity was calculated using the formula as follows:

$$\mathrm{Acidity}\;\%=\frac{Titervalue\times 0.1\;N\;NaOH}{\mathrm{Volume}\;\mathrm{of}\;\mathrm{the}\;\mathrm{sample}\;\mathrm{taken}\;\mathrm{for}\;\mathrm{titration}}\times \frac{Volumemadeup}{\mathrm{Weight}\;\mathrm{of}\;\mathrm{sample}}\times \frac{Equivalentweight\;of\;citric\;acid}{1000}\times 100$$

### 4.6. Measurement of the pH of the Pulp

Pulp of melons was homogenized without adding water, and pH was measured after calibrating the pH meter for pH 4 and 7 using standard solutions. Further, from each sample three readings were recorded, and such readings from triplicate samples were considered for taking the average and SD

### 4.7. Measurement of Total Sugars

Total sugar was measured using the phenol sulfuric acid method [40]. Approximately 1 g of fresh fruit sample was weighed and crushed with a pestle and mortar to a fine paste. The same was carefully transferred into a 15 mL falcon tube and centrifuged at 10,000 rpm for 5 min, and the volume of the supernatant was recorded. A 1.0 mL aliquot of sample was mixed with 0.5 mL of 5% aqueous solution of phenol in a test tube. Subsequently, 2.5 mL of concentrated sulfuric acid (85%) was added rapidly to the mixture. After allowing the test tubes to stand for 10 min, they were vortexed for 30 s and placed in a water bath for 20 min at room temperature for color development. Then, light absorption at 490 nm was recorded on a spectrophotometer. Reference solutions were prepared identically, except that the 2 mL aliquot of sample was replaced by double-distilled water. The phenol used in this procedure was redistilled, and 5% phenol in water (*w*/*w*) was prepared immediately before the measurement.

### 4.8. Measurement of Reducing Sugars

Reducing sugar in melon samples was measured using the modified Somogyi and Nelson method using a micro-titer plate [41,42] by reducing the reagent quantity. Sodium potassium tartrate tetrahydrate (62 g), sodium carbonate (12 g), sodium bicarbonate (8 g), and sodium sulfate (77 g) were dissolved in deionized water and diluted to 400 mL to make stock Solution I. Copper sulfate pentahydrate (2 g) and sodium sulfate (18 g) were dissolved in deionized water and diluted to 100 mL to make stock Solution II. They were stored separately to prevent copper oxidation [41] (Somogyi, 1952). Four parts of Solution I and one part of Solution II were freshly mixed to make the working reagent before analysis.

The arsenomolybdate color reagent was prepared according to [42]. Ammonium molybdate (12.5 g) was dissolved in 225 mL of deionized water and mixed with 10.5 mL of concentrated sulfuric acid. Sodium arsenate dibasic pentahydrate (3 g) was dissolved in 12.5 mL deionized water and mixed with the ammonium molybdate solution. The reagent was incubated at 37 °C for 24–48 h and stored in a brown bottle.

Approximately 1 g of fresh sample was weighed and crushed with a pestle and mortar to a fine paste. The same was carefully transferred into a 15 mL tube and centrifuged at 10,000 rpm for 5 min, then the volume of the supernatant was recorded. The clear supernatant solution (45 μL) was placed in wells of a 96-well micro-plate (polypropylene, 360 μL well volume, flat-bottom, Corning Company, Corning, NY, USA) in triplicate. The working reagent (45 μL) was then added. The plate was covered with a micro-plate mat and taped with a layer of aluminum foil to prevent water vapor from leaving the plate during heating. The sample and reagent were mixed by shaking the plate on a micro-plate reader for 10 s (SpectraMax iD3 Multi-Mode Microplate Reader, Molecular Devices, CA, USA) and then heated in a boiling water bath for 20 min. The plate was placed on a rack in the water bath, and its bottom was immersed in the boiling water. After heating, the plate was placed on an ice pack and cooled for 5 min. The arsenomolybdate color reagent (45 μL) was then pipetted into each well. The plate was placed on the bench for 15 min to complete the color development. The absorbance was recorded at 600 nm within 40 min after adding the arsenomolybdate color reagent [43].

### 4.9. Measurement of Carotenoids

The total carotenoid content was measured using a spectroscopic method [44]. Initially, we conducted extraction using methanol (100%), ether (100%), and acetone (100% and 80% *v*/*v* in water). Based on the reproducibility and consistency of the results, 80% acetone was used for all samples.

Briefly, fresh pulp and whole fruit samples were weighed and transferred to a mortar. Then, 2–3 mL of 80% acetone (*v*/*v* in water) was added, and the sample was crushed into a smooth paste. Depending on the sample size, a few mL of 80% acetone was added along with 2 small spatulas of washed sand and further crushed to get complete extraction of the pigments. This was transferred into a 15 mL tube and centrifuged at 5000 rpm for 5 min at 4 °C. Spectroscopic analysis was conducted directly on the supernatant or after 1:1 dilution depending on the OD value (when the value was more than 0.9, samples were diluted). Optical density was recorded at 470, 643, and 666 nm, and the amount of carotenoids was calculated as per the authors’ formula.

### 4.10. Measurement of Ascorbic Acid

The ascorbic acid content in melon samples was measured by a spectrophotometric assay using DNPH reagent [45]. Briefly, 0.23 mL of 3% bromine water was added to 4 mL of centrifuged sample solution to oxidize the ascorbic acid to dehydroascorbic acid, and after that 0.13 mL of 10% (*w*/*v*) thiourea was added to remove the excess bromine. Then, 1 mL of 2,4-DNPH solution was added to formosazone. All standards, samples, and blank solutions were kept at 37 °C for 3 h in a thermostatic bath. After that, the samples were cooled in an ice bath for 30 min and treated with 5 mL chilled 85% H_2_SO_4_, with constant stirring. The absorbance was measured at 521 nm, and the amount in each sample was calculated using a standard curve of ascorbic acid.

### 4.11. Measurement of Total Polyphenols

Total phenolics of juice and fruit samples were measured using Folin–Ciocalteu reagent as described in our previous publication [31]. Briefly, a known amount of sample (flesh and whole fruit) was crushed and made into smooth paste without adding water or other solvent and centrifuged at 1000 rpm for 5 min at 4 °C. Then, a 50 μL sample was made up to 500 μL using distilled water, and these samples were mixed with 1.0 mL of 10-fold-diluted Folin–Ciocalteu reagent. To this, 0.8 mL of 7.5% sodium carbonate solution was added and mixed slowly. After the mixture had been allowed to stand for 30 min at room temperature, the absorbance was measured at 765 nm. Catechin was used as standard and using the standard curve, the total polyphenols in each sample were calculated, and results were expressed as mg g^−1^ fresh weight of samples.

### 4.12. Biological Activities of Different Samples of Melons

The following in vitro assays were carried out to estimate the contribution of melon phytochemicals towards biological functions. A known amount of edible flesh portion of melon was crushed into a smooth paste using a pestle and mortar placed on an ice pack, and the resulting semisolid material was centrifuged at 1000 rpm for 10 min at 4 °C. The clear supernatant was used for assay after dilution in different assay solvents.

#### 4.12.1. DPPH Assay

The DPPH radical scavenging assay was performed as per our previously published protocol [31]. Different concentrations (25, 50, and 100 μg mL^−1^) of melon juice prepared as mentioned in Section 4.11 and ascorbic acid were taken in test tubes, and the volume was adjusted to 100 µL using MeOH. Then, 2.5 mL of 0.1 mM methanolic solution of DPPH (OD_517_ adjusted to 0.9–1.0) was added to these tubes and shaken vigorously. The tubes were allowed to stand at 27 °C for 20 min. The control was prepared without any extract, and MeOH was used for the baseline correction. Changes in the absorbance of the samples were measured at 517 nm. Radical scavenging activity was expressed as the inhibition percentage, calculated using the formula mentioned below, and results were expressed as % scavenging activity.

$$\%\;\mathrm{Radical}\;\mathrm{scavenging}\;\mathrm{activity}=\left[\frac{\left[\mathrm{Control}\;\mathrm{OD}-\mathrm{SampleOD}\right]}{\mathrm{Control}\;\mathrm{OD}}\right]\times 100$$

#### 4.12.2. Nitric Oxide (NO) Induction Assay

The NO induction activity assay was performed using the spectrophotometric method using Griess reagent as explained in our previous publication [36]. Different concentrations (equivalent to 25, 50, 100, and 200 µg mL^−1^) of melon samples were added to 250 µL of sodium nitroprusside solution (25 mM), and the tubes were incubated for 2 h at 37 °C. Aliquots (125 µL) of the incubated mixture were transferred to 96-well plates and treated with 75 µL of Griess reagent (1.0% sulphanilamidein, 5% H_3_PO_4_, and 0.1% naphthylenediamine dihydrochloride). The chromophore absorbance formed during diazotization of the nitrite with sulphanilamide and subsequent coupling with naphthalene diamine dihydrochloride was immediately read at 570 nm using Spectamax iD3, multimode microplate readers (Molecular Devices, LLC. San Jose, CA 95134, USA). The absorbance of samples was compared with standard sodium nitrite treated with Griess reagents under similar conditions, and results were expressed as µM of nitrite formed.

#### 4.12.3. ACE Inhibition Assay

The ACE inhibition assay was performed using a spectroscopic method based on the protocol described by Hernández-Ledesma et al. (2003), with slight modifications. Briefly, 110 µL of the substrate hippuryl histidine lysine (HHL) dissolved in pH 8.3 buffer with 0.3 M NaCl and 25 µL of ACE dissolved in glycerol at 50% were added to 20 µL of melon samples [46]. The reaction was incubated at 37 °C. The ACE activity was stopped by the addition of 110 µL of 1 N HCl. The hippuric acid formed in the enzymatic process was extracted using 1 mL of ethyl acetate and separated by centrifuging at 3000× *g* for 10 min. Then, 750 µL of the organic layer was taken and dried at 85 ± 5 °C for 10 min. The dried residue was dissolved in 1 mL of distilled water, and the absorbance was measured at 228 nm. The reaction blank was prepared in the same way indicated above, except HCl was added before adding the enzyme. The determinations were carried out in triplicate. The sample blank was prepared in the same way as the reaction blank, replacing the sample with water.

$$\%\;\mathrm{ACE}\;\mathrm{inhibition}\;=\frac{\left(B-A\right)}{\left(B-C\right)}\times 100$$

where B is the absorbance with ACE and HHL without the ACE inhibitor; A is the absorbance with ACE, HHL; and C is the absorbance with HHL without ACE and sample/standard components.

### 4.13. Statistical Analysis

All the analytical and activity study experiments were conducted in triplicate independently, and the results are expressed as means ± SD. Statistical analyses were performed using ANOVA, and data were compared using Tukey’s post-test analysis in GraphPad Prism software version 5.00.288. Unless mentioned, values with *p* < 0.05 were considered significant, and those with *p* < 0.01 were considered highly significant. In order to understand the relationship between the content and activity, we also calculated correlation coefficients between primary nutrition, physical parameters, and secondary nutrients and biological activity. Additionally, for nutrition content, one-way ANOVA was done using a non-parametric test and post comparison using a Kruskal Wallis H test with IBM SPSS (Version 16.0). The information is included as supplementary data.

## 5. Conclusions

The results of the current study indicate that fruit and vegetable melon varieties, especially locally available landraces and wild varieties, have good nutritional properties that may benefit human health, allowing us to address chronic diseases through food and nutrition. Results indicate that among the landraces, Sidoota and Alper Green have shown the highest TSS (11.25–12.20 °B) content, and reducing sugar of 2.84 mg g^−1^ fresh weight was found in both Sidoota and Pappusa. This suggests these can serve as a good carbohydrate nutrition source. Other landraces, Mage Kaayi-2, and wild types Budamekaayi and Small melon reported high content of polyphenols, and most of the landraces exhibited good NO induction activity, ACE inhibition potential, and good content (33.6–38.6 of mg g^−1^ fresh weight). Small melon and Budamekaayi, two wild types, have shown good radical scavenging activity at 100 ppm. *Agrestis* and Small melon have also shown good NO induction activity at 100 ppm. More than 60% of samples used exhibited good ACE inhibition activity (>70% at 100 ppm), which was comparable with captopril. These results suggest that these varieties can also serve as a good source of traits for improving commercial melons by breeding for desired qualities such as appearance, organoleptic characteristics, and health benefits. Therefore, the varieties examined here can be used to improve human nutrition and to develop more nutritious melons. As there are very few detailed reports on secondary metabolites in this crop, identifying other secondary metabolites in these samples may provide a complete picture of their potential benefits. As many of these varieties are grown as cash crops in rural areas, developing improved varieties also supports small and medium-scale farmers.

## Figures and Tables

**Table 1:** Description and uses of melon accessions included in the study.

Type	Genotype	Botanical Group	Source of Germplasm	Use
Landraces	Kekkarale	*momordica*	Shivamogga district, Karnataka	Ripe fruits consumed with jaggery
	Kekkarike	*momordica*	Hassan district, Karnataka	
	Minake	*chandalak*	Chamarajanagar district, Karnataka	
	Alpur green	*indicus*	Kadapa, Andra Pradesh	Ripe fruits for table purposes and juice preparation
	Alpur red	*indicus*		
	Ganjam	*indicus*	Davanagere district, Karnataka	
	Pappusa	*reticulatus*	Kadapa, Andra Pradesh	
	Sidoota	*reticulatus*	Davanagere district, Karnataka	
	Wild melon	Intermediate form of *agrestis* and *kachri*	Arunachal Pradesh	Ripe fruits for cooking and pickling
	Mekkekaayi	Unknown	Bagalkot district, Karnataka	
	Giriyaala	Unknown	Gadag District, Karnataka	Immature fruits for salad and ripe fruits for cooking
	Sambar savathe	*acidulus*	Sirsi district, Karnataka	Ripe fruits for cooking
	Mage kaayi 1	*acidulus*	Mangalore district, Karnataka	
	Mage kaayi 2	*acidulus*	Shivamogga district, Karnataka	
	Yeresavathe	Unknown	Darawad district, Karnataka	Immature fruits for salad and ripe fruits for cooking
	Kachri Jaipur	*kachri*	Rajasthan	Ripe fruits for cooking
Improved	Kashi Madhu	*chandalak*	ICAR-Indian Institute of Vegetable Research, Varanasi	Ripe fruits for table purposes
	IC321334	*chandalak*	ICAR-National Bureau of Plant Genetic Resources, New Delhi	
	IC321348	*chandalak*		
	IC321367	Unknown		
	IC321356	*reticulatus*		
	Cantaloupe1	*cantalopensis*	unknown	
	Cantaloupe 2	*cantalopensis*	unknown	
	Honeydew	*inodorous*	unknown	
	IC321371	*indicus*	ICAR-Indian Institute of Horticultural Research, Bengaluru	
	Arka Sheetal	*flexuosus*		Immature fruits for salad
Wild/weedy	Putti kaayi	*acidulus*	Bijapur district, Karnataka	Ripe fruits consumed with jaggery
	Agrestis	*agrestis*	Rajasthan	Not used
	Budamekaayi	*kachri*	Tumkur district, Karnataka	Not used
	Small melon	*callosus*	Shivamogga district, Karnataka	Not used

**Table 2:** Physicochemical properties of different melon cultivar samples. Values of total soluble solids (°B) and titratable acidity (%) of the pulp. Values are expressed as mean ± SD of three independent analyses (*n* = 6–8).

Sl. No	Genotype	Type	TSS	Titratable Acidity
			Avg ± SD	Avg ± SD
1	Kashi Madhu	Improved	11.25 ± 1.05	0.10 ± 0.01
2	IC321334		10.00 ± 0.65	0.18 ± 0.08
3	IC321348		8.15 ± 0.84	0.23 ± 0.03
4	IC321367		12.00 ± 1.02	0.18 ± 0.01
5	Cantaloupe 1		11.10 ± 0.98	0.08 ± 0.07
6	Cantaloupe 2		11.6 ± 1.13	0.12 ± 0.01
7	Honeydew		9.05 ± 0.95	0.09 ± 0.03
8	IC321371		10.65 ± 0.57	0.16 ± 0.06
9	IC321356		12.05 ± 1.26	0.09 ± 0.08
10	Arka Sheetal		3.65 ± 0.18	0.26 ± 0.01
11	Kekkarale	Landrace	5.40 ± 0.68	0.24 ± 0.06
12	Kekkarike		4.0 ± 0.34	0.27 ± 0.01
13	Minake		7.0 ± 0.35	0.21 ± 0.02
14	Alpur green		11.25 ± 0.8	0.15 ± 0.16
15	Alpur red		9.0 ± 0.94	0.18 ± 0.07
16	Ganjam		6.0 ± 0.85	0.21 ± 0.11
17	Pappusa		9.15 ± 0.91	0.11 ± 0.12
18	Sidoota		12.20 ± 0.83	0.24 ± 0.03
19	Wild melon		4.10 ± 0.54	0.52 ± 0.02
20	Mekkekaayi		4.0 ± 0.34	0.42 ± 0.09
21	Giriyaala		3.2 ± 0.33	0.37 ± 0.09
22	Sambar savathe		3.7 ± 0.21	0.28 ± 0.02
23	Mage kaayi 1		3.50 ± 0.09	0.24 ± 0.02
24	Mage kaayi 2		3.80 ± 0.11	0.2 ± 0.02
25	Yeresavathe		4.05 ± 0.12	0.19 ± 0.06
26	Kachri Jaipur		3.40 ± 0.87	0.32 ± 0.02
27	Putti kaayi	Wild	4.2 ± 0.24	0.25 ± 0.02
28	Budamekaayi		3.40 ± 0.32	0.22 ± 0.01
29	Agrestis		3.5 ± 0.22	0.25 ± 0.04
30	Small melon		3.50 ± 0.41	0.22 ± 0.01

**Table 3:** Nutritional value of melon cultivar samples. Values for total sugars, and reducing sugar content (expressed as mg 100 g^−1^ of fresh weight (FW)). Values are expressed as mean ± SD of three independent analyses (*n* = 6–8).

Sl. No	Genotype	Type	Total Sugar Content	Reducing Sugar Content
			Avg ± SD	Avg ± SD
1	Kashi Madhu	Improved	52.13 ± 2.61	2.83 ± 0.14
2	IC321334		42.03 ± 1.07	2.44 ± 0.07
3	IC321348		20.70 ± 0.24	1.67 ± 0.01
4	IC321367		34.56 ± 1.22	1.84 ± 0.31
5	Cantaloupe 1		23.23 ± 0.36	1.54 ± 0.10
6	Cantaloupe 2		61.40 ± 1.52	2.33 ± 0.09
7	Honeydew		53.76 ± 0.74	2.67 ± 0.17
8	IC321371		33.10 ± 0.21	1.98 ± 0.11
9	IC321356		36.78 ± 0.06	1.79 ± 0.12
10	Arka Sheetal		27.28 ± 0.53	0.96 ± 0.18
11	Kekkarale	Landrace	40.14 ± 0.09	1.06 ± 0.04
12	Kekkarike		58.71 ± 5.05	1.25 ± 0.02
13	Minake		27.28 ± 0.18	1.24 ± 0.11
14	Alpur green		21.73 ± 0.27	1.99 ± 0.20
15	Alpur red		53.89 ± 0.33	1.65 ± 0.17
16	Ganjam		42.66 ± 0.15	1.58 ± 0.39
17	Pappusa		53.13 ± 0.77	2.81 ± 0.03
18	Sidoota		24.33 ± 0.39	2.84 ± 0.11
19	Wild melon		43.53 ± 1.87	1.17 ± 0.07
20	Mekkekaayi		48.38 ± 1.46	1.38 ± 0.08
21	Giriyaala		29.54 ± 0.53	1.03 ± 0.01
22	Sambar savathe		55.29 ± 1.10	1.00 ± 0.29
23	Mage kaayi 1		36.78 ± 0.42	1.23 ± 0.02
24	Mage kaayi 2		31.67 ± 0.53	1.22 ± 0.09
25	Yeresavathe		31.40 ± 0.59	0.98 ± 0.01
26	Kachri Jaipur		25.49 ± 0.12	1.19 ± 0.05
27	Putti kaayi	Wild	46.52 ± 0.45	1.64 ± 0.18
28	Budamekaayi		25.42 ± 0.36	1.00 ± 0.04
29	Agrestis		54.16 ± 0.33	0.98 ± 0.03
30	Small melon		28.78 ± 0.56	0.68 ± 0.13

**Table 4:** Nutritional value of melon cultivar samples. Ascorbic acid (mg 100 g^−1^ of fresh weight (FW)) and total polyphenols (mg 100 g^−1^ FW). Values are expressed as mean ± SD of three independent analyses (*n* = 6–8).

Sl. No	Genotype	Type	Ascorbic Acid	Polyphenols
			Avg ± SD	Avg ± SD
1	Kashi Madhu	Improved	33.81 ± 0.29	11.11 ± 0.39
2	IC321334		31.52 ± 0.35	13.26 ± 0.60
3	IC321348		33.85 ± 0.08	10.84 ± 0.64
4	IC321367		33.805 ± 0.39	11.79 ± 1.07
5	Cantaloupe 1		37.21 ± 1.59	11.80± 0.36
6	Cantaloupe 2		33.42 ± 0.05	14.56 ± 0.58
7	Honeydew		31.97 ± 0.29	5.99 ± 0.32
8	IC321371		36.18 ± 0.55	13.88 ± 0.85
9	IC321356		32.55 ± 1.00	14.07 ± 1.19
10	Arka Sheetal		33.25 ± 0.35	5.39 ± 0.62
11	Kekkarale	Landrace	32.29 ± 0.66	8.42 ± 0.21
12	Kekkarike		31.90 ± 0.53	7.87 ± 0.28
13	Minake		34.12 ± 0.502	9.51 ± 0.55
14	Alpur green		36.94 ± 0.53	16.25 ± 1.46
15	Alpur red		33.44 ± 0.344	12.90 ± 0.69
16	Ganjam		32.75 ± 0.82	8.416 ± 0.68
17	Pappusa		35.04 ± 0.87	9.77 ± 0.87
18	Sidoota		33.80 ± 0.66	13.30 ± 0.58
19	Wild melon		36.71 ± 0.38	15.32 ± 0.53
20	Mekkekaayi		36.87 ± 0.61	14.43 ± 1.50
21	Giriyaala		34.90 ± 0.35	9.82 ± 0.56
22	Sambar savathe		38.59 ± 0.48	4.84 ± 0.44
23	Mage kaayi 1		31.90 ± 0.85	7.92 ± 0.55
24	Mage kaayi 2		32.01 ± 0.18	22.03 ± 0.29
25	Yeresavathe		34.71 ± 1.29	9.87 ± 0.65
26	Kachri Jaipur		36.85 ± 0.63	17.92 ± 0.54
27	Putti kaayi	Wild	33.62 ± 0.02	8.57 ± 0.33
28	Budamekaayi		38.63 ± 2.17	18.70 ± 0.51
29	Agrestis		34.26 ± 0.56	11.42 ± 0.48
30	Small melon		38.25 ± 0.98	18.68 ± 0.59

**Table 5:** Radical scavenging activity of melon cultivar samples as measured by 2,2-diphenyl-1-picrylhydrazyl (DPPH) scavenging assay. Values are expressed as means of three independent analyses at each tested concentration (*n* = 6–8). The SD was less than 5% of the mean. * Significant (*p* < 0.05) and ** highly significant (*p* < 0.01) compared to control and standard at lower concentrations.

Sl. No	Genotype	Type	Con in μg mL^−1^		
			25	50	100
1	Kashi Madhu	Improved	19.57	49.36	89.57 *
2	IC321334		57.59	75.8	86.90 *
3	IC321348		17.78	25.13	37.5
4	IC321367		45.27	42.67	52.04
5	Cantaloupe 1		8.48	14.38	29.08
6	IC321356		51.69	79.1	89.94 *
7	Cantaloupe 2		30.27	59.58	56.03
8	Honeydew		17.69	30.92	52.62
9	IC321371		38.77	65.39	74.76
10	Arka Sheetal		1.12	4.29	7.14
11	Kekkarale	Landrace	12.98	18.55	25.96
12	Kekkarike		28.51	38.94	47.87
13	Minake		6.65	12.66	24.79
14	Alpur green		10.82	18.56	21.52
15	Alpur red		21.26	47.42	66.62
16	Ganjam		19.85	23.23	30.46
17	Pappusa		17.85	37.85	53.54
18	Sidoota		0.15	2.62	19.54
19	Wild melon		8.77	16.28	33.63
20	Mekkekaayi		44.47	70.85	97.66 **
21	Giriyaala		35.32	63.02	90.96 **
22	Sambar savathe		14.86	15.14	26.43
23	Mage kaayi 1		34.92	44.77	58.77
24	Mage kaayi 2		67	75.77	88.14 *
25	Yeresavathe		13	25	43.14
26	Kachri Jaipur		52.18	78.65	82.27
27	Putti kaayi	Wild	16.2	13.73	32.3
28	Agrestis		33.96	37.45	57.8
29	Budamekaayi		53.06	69.29	79.9
30	Small melon		63.73	78.15	89.76 *
31	Ascorbic Acid	Standard	85.25	90.57	94.23

**Table 6:** Nitric oxide (NO) induction activity of melon cultivar samples. Values are expressed as % increase in nitrite formed compared to blank and expressed as means of three independent analyses at each tested concentration (*n* = 6–8). SD was less than 5% of the mean.

Sl. No	Genotype	Type	Concentration (μg mL^−1^)			
			25	50	100	200
1	Kashi Madhu	Improved	71.14	82.25	83.98	86.34
2	IC321334		67.22	69.77	73.23	83.7
3	IC321348		12.34	15.34	55.67	65.23
4	IC321367		47.38	52.94	67.59	72.05
5	Cantaloupe 1		9.88	33.38	43.1	63.5
6	IC321356		14.98	41.65	67.41	72.23
7	Cantaloupe 2		10.7	15.61	64.86	67.68
8	Honeydew		68.13	78.51	79.79	86.25
9	IC321371		18.25	32.91	58.31	65.04
10	Arka Sheetal		60.67	61.68	68.32	70.78
11	Kekkarale	Landrace	57.58	61.31	66.68	83.61
12	Kekkarike		8.33	14.79	68.23	69.78
13	Minake		14.16	26.72	61.68	68.32
14	Alpur green		24.8	49.93	63.4	77.6
15	Alpur red		50.38	69.23	74.6	83.16
16	Ganjam		67.41	69.68	82.25	85.61
17	Pappusa		56.84	75.05	79.88	86.43
18	Sidoota		15.87	43.1	61.03	78.6
19	Wild melon		8.32	14.78	68.23	69.77
20	Mekkekaayi		63.04	68.05	72.23	74.87
21	Giriyaala		67.22	74.14	80.43	83.7
22	Sambar savathe		23.43	42.82	60.4	62.85
23	Mage kaayi 1		57.57	61.31	66.68	83.61
24	Mage kaayi 2		47.38	57.12	65.68	66.68
25	Yeresavathe		67.22	69.68	74.43	84.89
26	Kachri Jaipur		50.3	54.39	63.04	73.78
27	Putti kaayi	Wild	31.18	54.85	62.77	63.59
28	Agrestis		58.86	70.96	82.79	87.16
29	Budamekaayi		48.66	59.13	60.58	71.69
30	Small melon		30.44	66.68	74.33	82.34

## Supplementary Materials

The following are available online at https://www.mdpi.com/article/10.3390/plants10091755/s1, Table S1 Descriptive statistical analysis of nutritional value of melon samples; Table S2 Post Hoc analysis of variation using Kruskal Wallis H test; Table S3 Correlation between Content of nutrition and Biological activity of different melons analyzed
