# Supplementary material for: Nutritional Composition and Health Benefits of Various Botanical Types of Melon (Cucumis melo L.)
Source: Plants (Basel). 2021 Aug 24;10(9):1755. doi: 10.3390/plants10091755 (PMC8469201; doi:10.3390/plants10091755)
Supplement: Supplementary file 1 [file plants-10-01755-s001.zip › plants-1329806-supplementary.pdf]

Table-S1 Descriptive statistical analysis of nutritional value of melon samples

Average value	Use of melons (Number of samples)	Mean	Std. Deviation	Std. Error	95% Confidence Interval for Mean		Minimum	Maximum
					Lower Bound	Upper Bound		
Ascorbic acid	Fruit (18)	33.79	1.65	0.39	32.97	34.61	31.51	37.21
	Vegetable(9)	35.09	2.35	0.78	33.28	36.90	31.90	38.59
	Wild (3)	37.05	2.42	1.40	31.04	43.05	34.26	38.63
	Total (30)	34.50	2.15	0.39	33.70	35.31	31.51	38.63
Carotenoids	Fruit(18)	9.46	4.65	1.10	7.15	11.78	3.41	21.67
	Vegetable(9)	9.44	6.03	2.01	4.80	14.08	4.05	21.23
	Wild(3)	5.25	1.82	1.05	0.72	9.78	3.38	7.02
	Total(30)	9.03	4.96	0.91	7.18	10.89	3.38	21.67
Polyphenols	Fruit(18)	11.24	2.77	0.65	9.86	12.62	5.99	16.25
	Vegetable(9)	11.95	5.85	1.95	7.45	16.44	4.84	22.03
	Wild(3)	16.27	4.20	2.42	5.84	26.70	11.42	18.70
	Total(30)	11.96	4.17	0.76	10.40	13.51	4.84	22.03
Total Sugars	Fruit(18)	39.88	11.34	2.67	34.24	45.52	25.40	61.40
	Vegetable(9)	42.39	11.08	3.69	33.88	50.91	25.49	53.89
	Wild(3)	29.45	4.41	2.54	18.50	40.40	25.42	34.16
	Total(30)	39.59	11.12	2.03	35.44	43.75	25.40	61.40
Reducing Sugars	Fruit(18)	1.74	0.69	0.16	1.39	2.09	1.00	2.84
	Vegetable(9)	2.10	0.49	0.16	1.72	2.47	1.19	2.81
	Wild(3)	1.09	0.24	0.14	0.50	1.69	0.82	1.28
	Total(30)	1.78	0.66	0.12	1.54	2.03	0.82	2.84

Table-S2 Post Hoc analysis of variation using Kruskal Wallis H test

Dependent Variable	Type of melon application	Veg/ Fruit	Std. Error	Sig.	95% Confidence Interval	
					Lower Bound	Upper Bound
Ascorbic acid	Fruit	Vegetable	0.79	0.11	-2.93	0.32
		Wild	1.21	0.01*	-5.74	-0.76
	Vegetable	Fruit	0.79	0.11	-0.32	2.93
		Wild	1.29	0.14	-4.61	0.70
	Wild	Fruit	1.21	0.01*	0.76	5.74
		Vegetable	1.29	0.14	-0.70	4.61
Carotenoids	Fruit	Vegetable	2.02	0.99	-4.13	4.18
		Wild	3.09	0.18	-2.14	10.56
	Vegetable	Fruit	2.02	0.99	-4.18	4.13
		Wild	3.31	0.21	-2.60	10.97
	Wild	Fruit	3.09	0.18	-10.5	2.14
		Vegetable	3.31	0.21	-10.9	2.60
Polyphenols	Fruit	Vegetable	1.64	0.67	-4.08	2.66
		Wild	2.51	0.05*	-10.1	0.12
	Vegetable	Fruit	1.64	0.67	-2.66	4.08
		Wild	2.68	0.12	-9.83	1.19
	Wild	Fruit	2.51	0.05*	-0.12	10.18
		Vegetable	2.68	0.12	-1.19	9.83
Total Sugar	Fruit	Vegetable	4.45	0.57	-11.6	6.62
		Wild	6.79	0.13	-3.51	24.37
	Vegetable	Fruit	4.45	0.57	-6.62	11.64
		Wild	7.26	0.08	-1.96	27.85
	Wild	Fruit	6.79	0.13	-24.3	3.51
		Vegetable	7.26	0.08	-27.8	1.96
Reducing Sugars	Fruit	Vegetable	0.25	0.16	-0.87	0.15
		Wild	0.38	0.10	-0.14	1.43
	Vegetable	Fruit	0.25	0.16	-0.15	0.87
		Wild	0.41	0.02*	0.16	1.84
	Wild	Fruit	0.38	0.10	-1.43	0.14
		Vegetable	0.41	0.02*	-1.84	-0.16

Note: The mean difference is significant at the 0.05 level.* indicate values are significant for specific parameter compared to respective type.

Table-S3 Correlation between Content of nutrition and Biological activity of different melons analyzed.

**Activity →**	**DPPH assay**			**Nitric oxide inhibition (%)**				**ACE Inhibition**
**Conc. in ppm**	**25**	**50**	**100**	**25**	**50**	**100**	**200**	**100**
Ascorbic acid	-0.14	0.03	-0.02	-0.24	-0.10	-0.31	-0.27	-0.08
Carotenoids	-0.22	-0.10	0.03	-0.06	0.00	-0.06	0.11	0.13
Polyphenols	0.50	0.67	0.56	-0.18	-0.09	-0.17	-0.14	-0.04
